# From ‘Sensory’ to ‘Anxiety’: Embodied States, Relational Care, and Ethical Transformation in Parental Care for U.S. Neurodivergent Childhood

**DOI:** 10.1007/s11013-026-10018-z

**Published:** 2026-09-03

**Authors:** Elsa Davidson

**Affiliations:** Department of Political Science, Law, History, and Anthropology, Montclair State University, Montclair, New Jersey USA

**Keywords:** Disability care, Neurodivergent childhood, Sensory, Anxiety, Interembodiment, Intercorporeality, Practice and ethics of care

## Abstract

This paper draws on an ethnography of care for social–emotional differences in middle-class North American childhood to explore the relation between care practices employed by parents of neurodivergent, or neuroatypical, children, and emergent ethics of care. Focusing on a single case of asymmetrical care, that between a mother and a neurodivergent child, I draw on video-recorded interviews as well other ethnographic material to highlight how practices of care emphasizing forms of bodily and emotional connection, and labors of attunement to embodied states and forms of expression, enable shifts in the caretaker’s perception of, and affective orientation toward, embodied states. In doing so, I show how moments of partially shared feeling across bodies (interembodiment) and intercorporeal connections can engender the parent–caretaker’s perception of enhanced intersubjective understanding, and authentic insight into the child’s embodied experience, particularly with regard to anxious and excited states. As such, I describe the caretaker’s perceptual shift from an individualizing evaluation of her child’s “sensory issues” to a relational understanding of “anxiety” as a collaboratively engendered embodied emotional state that can be altered, felt, shared, and alleviated through non-verbal tactile communication. In turn, I argue that it can be seen as a “gift” of ethical self-transformation that has implications for the care of neurodivergent childhood. In doing so, I suggest that such insight engenders care for a neurodivergent “other” that may come closer to preserving the other’s singularity. Moreover, I argue that such insight rejects a stereotyped understanding of embodied neurodivergent selfhood as bounded, brain-based, and impermeable.

## The “12-Second Hug”

Sitting in front of a Zoom screen during the summer of 2021, I listened as V*, a white college professor and mother to nine-year-old Sam*, recalled care strategies she had used to defuse moments of crisis and conflict at school, home, and the playground. Sam, whom V described as “neuroatypical”, had struggled during the semi-quarantine of the Covid-19 pandemic. As we talked, she mentioned one such strategy: enveloping Sam in a long hug with deep pressure, which she had found calmed him when he was upset. “We used to do like a 12-second hug, cause I thought it would calm him down and he needed—I thought of it in terms of ‘sensory,’” she said. Well into Sam’s elementary school years, “sensory” was for V a mental shorthand for Sam’s differences. She also used it to reference this haptic approach, a form of soothing through pressured touch that, like headphones in hectic and noisy environments, she had noticed defused his agitation. Over the years, however, V reported that she had come to think differently about her son’s keyed-up moments and “meltdowns” (as she called them), and what he might need or prefer at such times. Listening to her narrate her caregiving experience, it became apparent that her experimentation with practices of care had transformed her understanding of his embodied experience. So had her keen attunement to his embodied states (e.g., sleeping, awake, trying to sleep, sick, hungry, sad, angry, energetic). Moreover, these experiences provoked reflection on her own embodied and affective experiences of caretaking.

This article draws from a New York City-based ethnographic research project investigating how middle-class parents care for “neurodivergent” children’s embodied experiences and capacities. Centering one mother’s particular experiences and accounts of caretaking, it offers a micro-level, narrative-focused examination of practices of parental care for neurodivergent childhood. Mindful of the ethical potential inhering within “asymmetrical” relations of care (Mattingly & McKearney, 2023), I examine in what follows V’s descriptive narrations of her caretaking strategies and experiences. As such, I underscore the potential of efforts to attune to the embodied state of a cared-for “other” to provoke shifts in practice and perspective. In doing so, I link such shifts to V’s experiences of “*interembodiment*” (Bunkley, 2022: 258), or shared states across bodies, and moments of intercorporeal connection between V and her son. Moreover, I consider V’s narrations of self-transformation, focusing particularly on how transformations in care and perception have “unsettled” and reworked her sense of what “good care” for Sam specifically requires (cf. Arnold & Black, 2020; Cook & Trundle, 2020).

My examination of such care practices centers V’s narrations of care for Sam’s embodied and affective experience of distress in particular—what is often called by parents and others, borrowing from a clinical vernacular, “emotional dysregulation.” As such, I relate her representations of attuned observing to a shift in her perception of his acutely anxious and intensely excited states, and how best to care for him during these moments. Further, I consider the role of their shared embodied states, and moments of intercorporeal connection in this perceptual shift. As her self-reflective descriptions reveal, these experiences entailed her affective reorientation toward her son’s embodied and emotional experience, such that she felt their connection to one another was enhanced. In turn, this empathy-inducing experience has shaped the care she has provided over time. Moreover, it has supported her jettisoning of her long-standing presumption of a causative relationship between Sam’s embodied anxious states and what she thought of as his atypical sensory–neurological makeup. I argue that V’s shift in perspective and the evolution of her care illustrate the ethical potential of parental care for children with social and emotional differences—often labeled “neurodivergent” or “neuroatypical” in everyday public discourse. In so doing, I stress the importance of shared embodied experience, intercorporeal connection, and attunement to the other’s embodied states in fomenting shifts in parental perspective on children’s neurodivergent embodied experience. At the same time, I foreground the role of engagement with neurodiversity and care-focused media and expertise in engendering such shifts. As a consequence of their engagement in such caretaking practices, parents like V may come to newly assess and appreciate aspects of neuroatypical children’s embodied experience that they have found “hard to read”, or misjudged. In turn, such reappraisals may shape both care practices and insight into dominant conceptualizations of neurodivergent people’s affective experiences, including those of anxiety.

The analysis that follows is drawn from two wide-ranging, video-recorded interviews with V in 2021 and 2023, part of ethnographic data collection conducted in New York City intermittently between 2018–2023. This (IRB-approved) research has involved in-depth interviews with parents of neurodivergent children and also speech, play, and occupational therapists. It has also entailed observations of private “social skills” play therapy for neurodivergent children, and participation in online events focused on children’s social and emotional differences and services. I have additionally relied on publicly available archives of an online parent support listserv, as well as relevant local and national media. Lastly, it is also inevitably informed by my personal experience as a parent of children with IEPs in New York City’s public education system.

In its focus on parental narrative, my ethnographic analysis builds on explorations of narration during moments of caretaking (Mattingly, 2014). As such, it makes no attempt to represent the perspective of the cared-for child in this case. To be clear, in this parent-focused research, I did not meet or interview V’s child. Indeed, outside of my above-mentioned observation of therapeutic social skills classes during an earlier phase of research (observations not referenced in this paper), the broader research from which this paper is drawn has not entailed my direct engagement with neurodivergent children. In relation to this choice, I am mindful of not assuming and (mis)representing how neurodivergent children experience the care they receive. Instead, I seek to illuminate the complexity, experimentation, and ethical insight that can be associated with such parental caretaking. I also aim to reveal the capacity of such care to call into question doxic assumptions about care and neurodivergent embodied experience. Thus, I offer an interpretation of one mother’s embodied narration of her lived caretaking past. In focusing on a single person, this approach does not provide, or claim to provide, a representative examination of even middle-class parental care for neurodivergent children. Instead, it allows for an up-close illumination of how enactments of, and self-reflection about, care, and experiences of self-transformation, may shape a dynamic ethics of parental care. Moreover, it reveals some potentials of care-focused ethical transformation in the particular context of the late liberal USA. At the same time, this approach spotlights the affective experience of forms of embodied and corporeal convergence on the caretaker, as well as uncertainty and experimentation associated with parental care for childhood disability/difference.

## Neurodivergent Childhood, Parental Care, and Ethical Potentials of Care

As anthropologists and others have argued, globally rising rates of diagnosis of forms of neuroatypicality in childhood such as autism and ADHD can be attributed to reorganizations in diagnostic criteria (Grinker, 2007; Mattingly, 2017). This uptick has engendered new biosocial identities and communities in the late twentieth and first decades of the 21^st^ century. It has also sparked critical analysis of identity and experience within ableist contexts, spearheaded and maintained by autistic self-advocates and their allies (Grinker, 2007; Hacking, 1999; Hart, 2014). In turn, the emergence of such biosocialities and communities has been grist for anthropological analyses examining the maintenance and transformative potentials of neurodivergent identities and socialities (e.g., Ginsburg & Rapp, 2024). For example, beginning in the late 1990s, the Ethnography of Autism Project at the University of California at Los Angeles, investigated social interaction and meaning-making among autistic children and their families. Focused on familial household contexts, this work has enriched understandings of autistic sociality and means of its facilitation through care practices (Ochs et al., 2004, Ochs & Solomon, 2010; see also Fein, 2020). Moreover, anthropologists investigating intersections of kinship, care, and disability over the past two decades have made critical contributions to understandings of parent–caretakers’ practices of care and advocacy for childhood disability including forms of “invisible disability” such as autism and ADHD. Exploring contexts within and beyond the familial household, such work has highlighted dynamics emergent within the caretaking of neuroatypical children, including negotiation of “mother blame” (Blum, 2015; Landsman, 2009; Singh, 2004). It has also investigated parental advocacy in relation to dynamics of medicalization and ableism (and in some scholarship, their articulations with systemic racism) that affect disabled children in medical, clinical research, and educational contexts. Further, such work has traced the emergence of intimate publics in the context of disability advocacy and care (Hart, 2014; Prince-Hughes 2011; Rapp and Ginsberg 2011, 2011, 2020; Ginsburg & Rapp, 2024; Blum, 2015; Manago et al., 2017; Mattingly, 2017; Oh, 2023). My analysis builds on this body of work. It also draws inspiration from ethnographic scholarship of parent–caretakers’ dynamic perceptions and translations of neuroatypical and otherwise disabled children’s embodied experience and affects (Jackson, 2020; Landsman, 2009; Mattingly, 2017; Rutherford, 2020), and their facilitations of children’s forms of communication and sociality (Hart, 2014; Ochs & Solomon, 2010; Rutherford, 2021).

At the same time, my account takes up concerns raised by scholars of feminist ethics and practices of care (Mol et al., 2010; Kittay 2020; Tronto, 1993) and in phenomenological anthropology. Such work has emphasized the ethical authority of vulnerable subjects, and the ethical possibilities inherent in asymmetrical relations of care (Taylor, 2010; Gottlieb 2002; Mattingly & McKearney, 2023; Mattingly and Throop 2018). As such, it has often foregrounded ethical insights and dilemmas of parents and others engaging in care for disabled children (op cit. Mattingly, 2014, 2017, 2019; McKearney, 2020). This has included consideration of the ethical transformation of the caretaker, as parents come to see their disabled child as the embodiment of a self-transformational “gift” (Kittay 1000; Landsman, 2009). In a related vein, some anthropologists have centered ethical and communicative potentials of intercorporeal relationality between carer and subject of care, and multimodal forms of communicative care (Jackson, 2020; Rutherford, 2020, 2025). For instance, some investigations of familial as well as therapeutic contexts have emphasized the role of multimodal, including haptic, intercorporeal forms of care in engendering intersubjective understanding in relations of care, however partial those relations may be (Csordas, 2008; Deslarlais and Throop 2011; Goodwin, 2017; Goodwin & Cekaite, 2018; Throop, 2012).

My own investigation of parent–child relations of care has revealed middle-class North American urbanites’ care of neurodivergent children to be experimental, morally uncertain, and perceptively dynamic. Parents I have interviewed, V among them, sometimes struggle to understand their children’s desires, feelings, and forms of embodied communication. In addition, their descriptions of care reveal consistent “tinkering” with caretaking strategies (Mol et al., 2010) and navigation of neuronormative environments and expectations on behalf of children. These struggles and forms of tinkering are in evidence in the following analysis of an embodied narration of care. V’s recounting of labors of attunement and reworking in her caretaking process over time, and of moments of interembodiment and intercorporeality she experienced, exemplify, I suggest, the ethical promise of asymmetrical relations of care. This argument places my account in conversation with scholarship examining the ethical transformation of care-providing subjects, including disability care dynamics (Cook, 2020, Gottlieb, 2002; Kittay 1999, Landsman, 2009, Jackson, 2020, Taylor, 2010; McKearney, 2020). Moreover, I engage anthropological insights about how haptic and other parental care strategies can foment sociality and forms of shared experience that may impact care (Goodwin, 2017; Goodwin & Cekaite, 2018; Jackson, 2020). At the same time, I follow others (op cit. Kittay and Landsman) in emphasizing the ethical “gift” of caretaking. Hence, I maintain that V’s practice-related reworking of her own understanding of her son’s anxious embodied states constitutes a kind of *political* gift, one that resists doxic understandings of neuroatypical anxious states and reactions that, in their alignment with neuroreductionist interpretations of difference, may not adequately represent lived, embodied experience (cf. Dumit, 2003; Martin, 2009; Mattingly, 2019; Rose & Abi-Rached, 2013; Vidal & Ortega, 2017; Wolf-Meyer, 2020). In turn, I suggest that V’s evolved understanding of her son’s embodied states indicates a refusal of autonomous and bounded conceptions of selfhood in a late liberal North American context (Obadia, 2020; Kittay 1999; Davy, 2019; Wolf-Meyer, 2020).

## An Uneven Urban U.S. Landscape of Parental Care for Neurodivergent Disability/Difference

In New York, as elsewhere in the USA, parental care for neurodivergent children is shaped by unequal and differential access to diagnosis and supportive care by race (Blum, 2015; Ginsburg & Rapp, 2024; Mandell et al., 2009). New York City’s public school system, the largest in the country, is also beset by overall persistent gaps in support for students with disabilities. Further, it has been repeatedly cited for inadequate care of children with disabilities prior to and during the pandemic (Public Advocate Report 2017; Zimmerman, 2021). For affluent New Yorkers, these circumstances often provoke reliance on private educational support services. These may include dependence on private occupational therapy and speech services, and use of private neuropsychological evaluations. Additionally, those who can afford to pay for private-pay mental health care with desirable pediatric providers who typically do not accept health insurance. They may also opt for expensive specialized private K-12 special education. Such privileged circumstances of care stand in contrast to those of children (and families) who, despite the possession of an IEP with stipulated mandated services, must rely on a patchwork of unevenly available providers and services within public school system (cf. Macpherson et al. 2023).

In addition, parental and educational care for neurodivergent childhood in the USA now articulates with a national turn toward social and emotional skill (Bialostok & Aronson, 2016; Fein, 2015; Gagen, 2015; Hoffman, 2009). In line with neoliberal reforms emphasizing public school accountability, this educational biopolitics increasingly entails documentation of children’s social and emotional development and difference (Davidson, 2020). In turn, this process intersects with extant dynamics of white privilege and the marginalization of students of color, who continue to experience unequal access to resources and segregation within the public school system (Cohen, 2021). As a result, such students may experience daily forms of debilitation within public educational contexts and be less likely to access supportive disability recognition (Puar, 2017; Shildrick, 2019).

Within this uneven context, neurodiversity discourse and neurodiversity-sensitive care shape orientations toward care for middle-class parents (V, as we will see, is no exception). Moreover, familiarity with an ethos of neurodiversity celebration is manifest among special educators, school administrators, and a range of private therapeutic providers that offer support services to neurodivergent children. Despite this, and in line with the findings of interdisciplinary scholarship of parental disability care, my interviews reveal parents’ frustration with ableism experienced by their children. They also feature parents’ critiques of school inclusion as practiced in public schools (cf. Reeves et al., 2022), and articulations of commitment to the recognition of human neurodiversity (cf. Hart, 2014). Such commitments are experientially rooted; my interview data, as well as archived listserv posts seeking support for children with “invisible disabilities,” reflect parents’ frequent preoccupation with educators’ comments about their children’s embodied differences. They also reveal deep concern with children’s social experiences at school, which, in turn, can motivate for those able to afford it investment in private, therapeutic “social skills” enhancement.

## A Landscape of Care: Privilege, Insecurity, and Ableism in Pandemic New York

V and her family occupy a position of racial and relative class privilege and do not experience structural racism and ableism as intersecting forces of debilitation (Puar, 2017). These privileges notwithstanding, V is a renter in an expensive city and housing market. In our interviews, she notes on multiple occasions the expense of services for Sam, or the absence, due to its cost, of a class or service that might be helpful. Lack of federal, state, and local public investment in infrastructures of care for neuroatypical and disabled children shape her approach to Sam’s care. For example, she avoids the cost of a private “neuropsych” evaluation for Sam, but is an avid consumer of podcasts and books about care for neurodivergent children (V recommended sources during both our video interviews), and mentions in passing her searches at the local public library for appropriate stories of neurodivergent childhood for Sam. Hence, her narrative reveals her attunement to a dominant neurodiversity ethos in her social milieu, and also chronicles her navigation of an uneven patchwork of forms of disability care for children available through New York City’s public education system (cf. Ginsburg & Rapp, 2024). Anemic public investment in infrastructures of care supportive of child disability within and beyond educational contexts compound this burden, and made Sam, V and their family particularly vulnerable to interruptions in care posed by pandemic disruption.

At the time of our first interview, in 2021, V and her family were attempting to regain their footing, piecing back together some normalcy after the period of school shutdown and life in the semi-quarantine of the Covid-19 pandemic. She was living in a 1.5-bedroom apartment in Brooklyn, New York, with her husband, Sam, and his little brother. She described Sam as social, into video games and making comic books, with a burgeoning interest in magic tricks. He didn’t have a formal diagnosis, which V attributed to the cost of private neuropsychological evaluations, but did have an Individualized Education Plan (IEP), granting him recognized disability status and services in school. V described cobbling together, since Sam’s kindergarten year, what felt like appropriate IEP services (e.g., speech, in-school counseling, ICT), in response to his atypical reactions and sensitivities to various environments at two different elementary schools he had attended. At the height of the pandemic, V had been chairing her department from a desk in their shared living space. She and her partner had little money for extra expenses during this period and both kids were attending school on Zoom. In the course of this interview, V smiled as she recalled a mouse problem in her apartment during this time. Retrospectively, this detail stands out in her narrative as emblematic of her sense of everyday conditions of life and parenting as beyond her control in her small apartment during the pandemic.

Sam’s support services were only intermittent during this time. Gaps in citywide services meant no services if a provider went on maternity leave, as Sam’s therapist did—a disruption that V reported had greatly upset him. V relayed being witness to Sam’s growing distress at the rupture in his life caused by being cooped up at home during the pandemic, and at times, Sam’s stress, and V’s situation caring for him under such constraints felt extremely difficult. V described, for example, moments during which Sam left the apartment and sat, in a state of acute distress, on the steps inside their building, making V worry about noise and bothering neighbors. A few times, he threatened self-harm. He also had trouble sleeping.

Such chronic and acute conditions shape the material and affective atmosphere of everyday life for many mothers of neuroatypical children I have interviewed prior to and during the pandemic (cf. Vincent, Oliver, and Paulopoulou 2023). Within this citywide and immediate, familial context, and even before she knew that he was neuroatypical, V sought to learn about Sam. She worked to facilitate his thriving at home and in school and community settings. She addressed his ongoing challenges and moments of acute suffering, and tried to attune to his needs and desires. Below, I describe V’s representation of practices of observation and attunement as a caretaker, and specific enactments of care she undertook within and beyond the family home. I then turn to her descriptions of her attempts to soothe Sam in times of acute distress, including experimental, and sometimes interembodied and intercorporeal, care. And, in relation to these narrations of care, I emphasize V’s own self-reflective analysis of the effects of her approach on herself and Sam. In turn, drawing on arguments developed particularly within phenomenological anthropology and the anthropology of care, I identify particular ethical potentials of the kinds of care she describes, within and beyond their own caretaking/care-receiving dynamic.

## Observational Attunement and “Tinkering” Care

When L was a newborn, V had Googled, “Is it normal for a baby to cry 23 hours a day?” And as time went on, the brief parental phase of sleeplessness associated with having an infant stretched into years. For years, L required hours of patting in bed every night. Telling me this, V demonstrated the patting gesture and recalled, rolling her eyes and laughing: “It was horrible…He would have *horrible* meltdowns if we tried to put him back in his bed.” Sam’s sleeplessness generated a caretaking habitus; V’s desire for them both to sleep set in motion a practice of close observation of Sam’s bodily signals. It also galvanized a care-focused research habit (V frequently listens to podcasts, and reads articles and books relevant to Sam’s care).

Both video interviews reveal the striking volume of V’s observations about Sam’s embodied feelings, movements, and non-verbal signals. “I could feel him buzzing with energy…tossing and turning,” V recalled of his struggle to sleep in pre-school and early elementary school. She noted a “cyclical” pattern wherein Sam’s body would shake and be “out of control” or “clumsier” during times of stress or excitement, and would behave differently when calm. In our first interview, V’s recollections of Sam’s early childhood linked his mood, affect, and actions to exposure to noises on city streets, bus rides, lack of sleep, an urge to urinate, or transitioning between activities. V remembered, for example, how Sam would clap his hands over his ears at motorcycles roaring past, and how, for years, they avoided birthday parties because of the possibility of popped balloons.

During our second Zoom video interview in 2023, V remained attentive in her recollections to Sam’s embodied states and forms of awareness. She also noted her recent, increasing attunement to signs of potential dysregulation. For example, she cited his “constant eating” after school, and insistent “requests for video games.” She also worried about a lack of personal boundaries, such as his tendency to invade others’ personal space concerned her, or ask (in her view, inappropriately) for other people’s food. Such attunement to Sam’s moods and actions was for V galvanizing. It prompted her to look for clues to help her understand potential triggers for dysregulation (e.g., hunger, being on the verge of sickness, arguing with a friend at school). V’s narration over the course of both interviews made clear her efforts to decipher possible bodily or social causes for embodied distress or excitement. She also described in detail her practice of modifying her care practices to improve care. Collectively, these enactments of care recall Annemarie Mol’s, Ingunn Moser’s and Jeannette Pols’ (2010), exploration of ethical care as locally constituted practice. Such practice, they note, entails “practical tinkering,” or “attentive experimentation” (p. 13)—phrases that accurately denote much of V’s chronicling during both interviews of the care she has provided her son. She attended to Sam’s lack of comfort with unstructured time with temporal solutions. She made color-coded lists of things for him to do depending on his mood. She drew calendars visually mapping out and structuring his time, and reviewed them with him. In doing so, she attended especially to periods of unstructured time such as the beginning of summer vacation, which she knew would be a vulnerable period for him. She created visual signs to support his capacity to complete everyday tasks. In the hallway outside Sam’s room, she pinned a note listing possible causes of sleeplessness (“Hungry? Have to pee? Thirsty?”). After-school meltdowns prompted her to add more protein to Sam’s diet, a change that she noted had made a positive difference at school and at home. Noise sensitivity led her to introduce Sam to headphones and listening to music. “Now, he knows when he needs noise,” she noted, her inflection conveying a sense of accomplishment.

## Acute Distress, Intercorporeal Moments, and Interembodiment

Sometimes, painful moments have triggered for V a tweaking of care strategies. During our second interview in 2023, she reflected on her son’s behavior, comparing moments of acute distress when he was a little younger, during the height of the pandemic, to more recent “big meltdowns.” V said that during these more recent events, when Sam had been profoundly upset, he had expressed that feeling through mild embodied aggression, or threats of self-harm. In narratively recalling such events, V took care to precisely describe what had happened, and to compare episodes carefully to past similar occasions. As she spoke, her focus on accurately relaying the facts of these events highlighted her own struggle with them. Her speech slowing at this point on the video recording, she gazed to the side as she recalled exactly what had happened during these events:

“It’s happened four or five times this year, um… But my responses have changed too [pause]. For a while I was having a really hard…[pause] I mean I’ve *always* had a hard time, like, who wouldn’t have a hard time (laughs quietly)?”

Her voice rose as she asked this rhetorical question, and then, V raised her hands up and then lowered them slowly, as if in a meditation gesture. “One tactic I learned was not to react, so it took a lot of work to learn how to be kind of stoic.” But then, V smiled; there was a problem with this “stoic” strategy. “I got so good at [being stoic] that he started feeling, I realized, that he started feeling like I was withdrawing, and that would make him even *more* upset…Like, I’d be affectless (V moves her hands across the front of her body back and forth, as if to signal a blank slate) and he’d get really agitated, like, “why aren’t you…?” Or I’d even leave the room and that would get him really upset,” she recalled. “So, what I more recently have been trying to do,” she continued, “is acknowledge that he is in a lot of pain while also putting up boundaries.” V told me that she says calmly out loud at such times: “I don’t like it when you curse at me,” and asks: “Can I put my hand on you?” In this moment of our Zoom call, V embodied this thought, placing her own hand on her arm.

“Just sitting by him, and showing empathy, partly with my eyes [pause], and not, like, not talk[ing] that much...and that actually seems to calm him down much quicker… and I’ve realized he doesn’t want to feel abandoned in those moments. When I’m very stoic he feels abandoned, and he needs to know that big emotions are O.K…We’ve been working on that.”

A podcast—she thought it was the *ADHD Parenting Podcast*, which she described as “wonderful”—had taught her to break down her actions in such moments. She learned from it, as she put it, “*where* to look”, but also how to look: “Like, actually making your eyes, like, look connected.” Here, V paused, cocked her head to the side and laughed, and stared at a distant point. “I don’t know how to…” she said, and then stopped speaking and demonstrated craning forward and looking intensely] V smiled and explained: “Sort of showing empathy through your eyes.”

V also reflected that her style of touch, carefully attuned to Sam’s desire for tactile connection, has shifted to become more intentional: “He still loves hugging, he’s a hugger, but what I’ve started to do more is put my hand on his leg or ankle.” V described how, at times, when Sam has appeared to V to be experiencing extreme panic, she has combined the two practices, making wordless eye contact with Sam as, simultaneously, she lightly places her hand on his leg. Here, V’s narrative description of care particularly recalls Marjorie Goodwin and Asta Cekaite’s description of how affectively rich familial relations are co-created through haptic care, as well as moments when touch is used “in coordination with other semantic resources” (2018: 130), as when a parent employs both a hug and ‘creaky voice’ (Goodwin, 2017: 88) to soothe an upset child (cf. Throop, 2012). When we spoke of these moments of visual and haptic connection in the course of our second interview, V again brought up empathy, connecting these instances to her becoming “more empathetic.” Moreover, she noted an effect of her empathic response: it had shortened the length of her son’s episodes of acute distress and anger.

In the flow of our conversational interview, “empathy” seemed to mean for V that she had achieved deeper understanding of and appreciation for her son’s experience. As such, her observation regarding how this empathy came about underscores Thomas Csordas’s point that moments of intersubjective connection tend to occur concretely and materially (2008), and via moments of intercorporeal exchange. For V, the experience of touch and her deployment of an explicitly conscious manner of looking and seeking eye contact with Sam generated a “shared space” “…in which neither…was the creator” (Merleau-Ponty quoted in Jackson, 2020: 63; cf. Goodwin, 2017 and Meyer, Streeck, and Jordan 2017).

Hence, even as we acknowledge the “…irreducible asymmetry and instability of perspectives and experiences assumed in even the most mutually attuned, empathic and intimate of intersubjective encounters” (Desjarlais & Throop, 2011: 91), we can observe that in the space of such moments, V perceived shared embodied feeling between herself and Sam. And at such times, she felt herself become “more empathetic” and thus more able to attune to his state of being in the world at that moment (cf. Throop & Zahavi, 2020). In essence, V was describing a feeling of intersubjectivity, in which, via literal contact with Sam, she came closer to his point of view, and hence, shared understanding (Duranti, 2010). Intercorporeal interaction fomented empathic insight, which in turn transformed V’s responsivity to Sam, and appreciation for his unique experience, or singularity (cf. Jackson, 2020 and Mattingly, 2019).

At a few points in these two video interviews, V flips back and forth in her speech between subjects, or simply leaves unstated who she was talking about, Sam or herself. Whose exhaustion, whose distress? In the ephemeral moment of our conversational exchange, it remains unclear. This ambiguity makes sense; the work of attunement and tinkering toward better care in the intimate space of family life generates convergences of embodied, affective, and lived, daily experience that can manifest in speech. Sometimes V herself commented on a blurring and sharing of their embodied experience. Thus, not only did V explicitly note her and Sam’s similarity (e.g., labeling herself as also “neuroatypical,” and mentioning in passing her own under-the-radar “sensory issues”), she observed that “my anxiety peaks when his anxiety peaks.” Additionally, there were other moments of interembodiment evident in her narration about which she did not provide any meta-commentary. Both mother and son often couldn’t sleep at the same time, and both were simultaneously exhausted. Sam’s “big meltdowns” have left her with “depressive episodes.” Both found the visualized temporality of calendars blocking time into squares soothing; “I realize now he just needs visuals…I realize for myself too, I need a board like that,” V said, slipping from Sam to herself.

## From “Sensory” to “Anxiety”

V’s attunement and the practices of care she has developed and tinkered with over time also have a broader social context. Cultural messaging and expert knowledge have additionally shaped her evolving understanding of her son’s embodied experience and her enhanced empathy, V’s narrations of care in both video interviews often revealed the interplay between practices (of care) and seeking expertise. Difficult moments provoked her to research. As V recalled,

“I was feeling really desperate because I really like to have solutions to things. I was feeling like, ‘I know these meltdowns happen; I can see when they are about to happen. What am I doing wrong?’...It was around the time he was getting soooo upset. And I thought I was doing well ’cause I was being so stoic. And that was when I started to read, and also I listen to a lot of mental health podcasts.”

V repeatedly referenced podcasts such as *ADDitude Magazine*’s “*ADHD Experts Podcast”* and “*AT Parenting Survival for Kids With OCD and Anxiety,” as* well as other “mental health podcasts” that she didn’t cite specifically, but acknowledged she regularly listened to. She cited advice from these podcasts (e.g., don’t give children the impression that anger is bad, or the instructions to achieve a certain kind of attuned looking at the cared-for other), and referred to a range of other resources she had found helpful. These included two particular listservs for New York City parents of kids with disabilities, and a series of YouTube videos about children’s executive function struggles (with tips on how to handle daily issues, such as getting ready for school). In addition, she mentioned the reading she had done about “giftedness” and being “twice-exceptional”—strands of public discourse about neurodiversity that she thought might, in part, explain Sam’s ways of being. She also referenced therapeutic providers (intermittent and often not reliably available school providers, and her son’s private therapist, who employed techniques of cognitive behavioral therapy).

Hence, V’s daily enactments of care, inflected at times by experts’ tips and conceptual framing, have been multifarious and profoundly affecting. They have entailed (for V) partially shared or overlapping embodied experiences (of sleeplessness, exhaustion, emotional downswings, and visual representations of time and activity). They have required persistent and careful observation of Sam and herself. And experimenting with care has led to moments of intercorporeal connection in response to distress—e.g., sharing a hug, minimal but purposeful touch pending his permission, touch in combination with a particular kind of eye contact, calmly voiced declarations, and sometimes, silent looking. As I have already suggested, these strategies and experiences have helped engender for V a sense of increased empathic attunement and intersubjective connection. At the same time, examination of her care narrative also reveals how the dynamic, fluid, and culturally mediated space of parental caretaking has led to a consequential shift in V’s understanding of Sam’s embodied feelings in times of excitement or moodiness, and moments of great anxiety and “meltdown”. V remembered this shift as a kind of epiphany that she first experienced when he was in second or third grade. But her narrations of care in the video interview recordings reveal this insight to be something that recurred, reactivated from time to time in moments of care. Whereas before she had interpreted what she called his “cycles” of buzzing excitement, or his desire to hug her and others, or his moments of acute suffering or upsetness as a matter of “sensory” difference or “issues,” she had, through watching him and caring for him over time, come to see “anxiety” as a more accurate framing. Starting first with the examples of uninvited hugging with others who might not want to be hugged, and hugging after a fight, V explained:

…*I used to see it in very sensory ways, but increasingly I see it as emotional in that that’s how he expresses closeness… through hugging. Like if we’re in a fight, he’ll just, like, want a hug, and it’ll resolve the tension. I used to see it as physical touch. I thought of it in terms of the sensory, like he needs the sensory [input]. But as he gets older, I think of it in…‘whole person’ terms… I just don’t see it so black and white anymore…*

For V, the physical–sensory reading of these moments did not adequately account for the emotional aspect of his experience and embodied feeling. At this moment in our conversation, V interrupted her description of her observations of Sam to mention that she was influenced by the work of neuroscientist and emotion expert Lisa Feldman Barrett, and her concept of the brain’s “body budget” (wherein the brain makes predictive adjustments to facilitate a bodily action, or cope with all kinds of stimulation, from both internal and external sources, as needed). As V explained, “Part of the body budget is like the physical, but part of it is other things going on.” Here, “other things going on,” like “whole person terms,” seemed to refer more broadly to her son’s experiences, including his experience of the external, social world. She continued in a reflective mode:

*What I’ve learned from watching him grow, sometimes he’d be in a horrible mood because of something at school with a child at school…but sometimes I’d find out, ‘oh, he’s about to get sick’…When I thought of him as having sensory issues, which I did for a really long time, I missed all the anxiety, and there’s a lot of anxiety there, but if I over-focus on anxiety, I miss the physical...*

At other points in these interviews, V further underscored “anxiety.” For example, she said in reference to her son’s “buzzing” with energy: “It took me forever to realize it was anxiety,” and not “something wrong with his nervous system.” At another point, she remarked, “I actually increasingly think of him in terms of having anxiety.” What is the content of “anxiety” in these instances? In these self-reflective observations, “anxiety” is clearly distinguished from “sensory.” In explaining to me her transformed perception, she equated reading her son’s affect and actions as “sensory” in various situations—e.g., hugging after a fight—to seeing things as “black and white.” In the context of our discussion, this implied un-nuanced or overly-simple interpretation. At the same time, in describing this situation, she opposed “sensory” and “emotion,” and also to anxiety-generating external and bodily events. Anxiety thus emerged in her descriptions as embodied and emotional, and something that can be collaboratively produced and mediated with others through experiences in the world (e.g., a conflict with another child at school). She also framed it as something that might come about as a result of bodily feelings, like the feeling of coming down with a cold. This emergent awareness provoked reappraisal. V now saw that her former judgment—that moments when Sam seems extremely upset, or “buzzing” with excessive energy, or desiring to hug boiled down to sensory difference—did not sufficiently take into account his multifaceted emotional, social, and bodily experience in daily life. This prior judgment, then, had represented his embodied acts and affects too simply, in “black and white,” self-contained, and individualized neurosensory terms. Realizing this, she discarded an interpretation that, in the past, she had shared widely with others in the hopes of steering their approaches to care for him (e.g., with teachers at his school). In making sense of V’s reappraisal here, and her chosen discourse of “anxiety,” we should again acknowledge the interplay between her care practices and pervasive cultural discourses and forms of media she accessed. For example, one of V’s preferred podcasts had “anxiety” in its name, she told me that her son’s therapist used the language of “anxiety,” and the topic of childhood anxiety is prevalent in public discourse (cf. Haidt, 2024; Hughes, 2022) and common conversational fodder in V’s middle-class milieu. However, V’s recollections of her reappraisal, as in the above quotes, are connected to her keen attentiveness Sam’s reactions in moments of care and at other times, suggesting the primacy of everyday practice, experimentation, and attuned observation in engendering her reappraisal. In this sense, her interpretive epiphany, like her more recent realization that a “stoic” attitude was not effective support in moments of acute stress, recalls Cheryl Mattingly’s point, following Gadamer (2013), that understanding is generated through immediate, first-person experiences that both exceed and confound previously held assumptions (2017). 

Overcoming such assumptions is not necessarily easy. Discourses of sensory difference that are framed causatively in relation to neurodivergent children’s forms of embodied expression, movement, and affect are widely pervasive in the middle-class social context in which V is raising her family. Parents, teachers, pre-school directors, itinerant special educators, speech therapists, and occupational therapists I have interviewed often talk about social, emotional, and communicative differences in childhood as “sensory” in nature, or a matter of “wiring.” Moreover, this causative and yet opaque neurosensory imaginary, which locates socially meaningful differences in the brains of individual children, is also arguably useful to parents. As I have suggested elsewhere (Davidson, 2021), the very vagueness of “sensory” discourse in the context of neurodivergent childhood serves to reattribute agency to the “sensory.” It is thus directed away from neurodivergent children who often experience social and academic exclusion on the basis of perceived character and moral traits, and also away from parents (mothers in particular) who are often blamed for children’s differences and disabilities (Landsman, 2009; Singh, 2004). As with other parents I have interviewed, V reported seeking to influence Sam’s supportive care at school by translating his embodied experience in terms of sensory difference. In the competitive economy of school performance, which as noted above, now emphasizes social and emotional capacities, such an interpretation can have value as a kind of inoculation against moralizing judgments that often stigmatize neurodivergent children.

## The “Gift” of “Anxiety”

V’s epiphany—that Sam’s embodied acts, affects, and embodied experience in moments of acute distress, or during prolonged periods of keyed-up energy, constitute expressions of anxiety—has had practical effects on her caretaking. So has her recognition of that anxiety’s multifaceted and often relational causes. “Thinking with” anxiety has primed V to try learn as much as she can about the immediate and recent situations, embodied as well as social and external, that might be affecting her son (e.g., a difficult bus ride to school, arguing with a friend or feeling left out, or a feeling of physical discomfort) and engendering his visibly anxious state or terrible mood. As she put it, “we go through the list [of possible causes].” A focus on anxiety has also heightened her awareness of her son’s verbal cues and actions, both in moments of acute distress, as she experiments with multimodal techniques of care, and everyday situations. V gave the example of her son’s requests for video games, or returning again and again to the refrigerator for more snacks after school, as signs she’s learned to recognize as the ramping up of an anxious state. Her focus on anxiety, in fact, caused a tweaking of ordinary care in the form of a new rule (no asking for anything until a snack has been consumed) and a different approach to meals (increasing morning protein intake). In addition, this form of awareness has also sparked her deeper self-attunement to her own embodied feelings. It was when V described embodied signs of what she now saw as Sam’s anxious states that she commented, “my anxiety peaks when his anxiety peaks.” This meta-observation, in turn, called to mind an example of a bad effect of such contagious anxiety:

“So, like, I’m working on being really mindful of what I say, like, when I notice him eating too much, because it sort of triggers my anxiety, like ‘You’re eating too much, you’re gonna be really unhealthy’, that sort of thing. So I’ve tried to train myself, ‘No conversations about food ’cause you’re anxious and you’re not thinking rationally.”

V’s narrative here moves first from observation to a shared affect of anxiety and anxious speculation. It then shifts to her response to what she perceived to be Sam’s overeating, and her subsequent response to her own reaction, an amendment to care that entails “regulating” expression of her own anxious thoughts when talking to Sam. In recognizing her own tendency, V is involved in what anthropologist Cheryl Mattingly has called “narrative revisioning,” which she describes as “the activity of coming to see oneself in a new way, coming to reform one’s sense of possibility, and reframe one’s commitments” (Mattingly, 2014: 20). While parenting her son in her kitchen, V has made a commitment to care differently, based on a new understanding of herself that emerged through care.

As this ordinary example illustrates, V’s interpretive shift to anxiety, which incites recognition of anxious moments (not just Sam’s but also hers), can cause further tinkering with care. But the shift toward an explanatory frame of anxiety has also engendered other forms of ethical insight. By discounting the view that Sam’s embodied responses across environments and situations are explainable solely in terms of his neurosensory makeup, and reading them instead as produced through his bodily and social experience of being with others and in different environments, V is able to appreciate the singularity of his experience (Mattingly, 2019: 419). At the same time, her purposeful shift of focus toward emotional, social and bodily aspects of her son’s experience suggests her rejection of neuroreductionist “received wisdom” about human neuroatypicality. Such neuroreductive framing, often embedded within both medicalizing and neurodiversity-affirming discourses, attributes to and locates within the brain complex social phenomena, behavior, and embodied experience (Wolf-Meyer, 2020: 19–20; cf. Dumit, 2003, Martin, 2009, and Silverman, 2018). V’s first-person narration, then, tracks the undoing of her “unexamined prejudgments” (Arendt quoted in Mattingly, 2019: 423) of Sam’s embodied states, and her own emergent critical awareness of this doxic misunderstanding. This, in turn, brings her into closer alignment with his embodied state, allowing her to enhance the specificity and responsiveness of her care. Good care, then, can’t be judged “in general terms and from the outside, but [is] something to *do*, in practice, as care goes on” (Mol et al., 2010: 13).

## Sensory Difference, (Im)Permeable Selfhood, and Care’s Potential

Writing in a very different ethnographic context (an intentional community in the contemporary USA), Julienne Obadia has shown how, in late liberal North America, being together “in community” depends on a powerful commitment to, and belief in, individualized personhood. In this ideological context, “healthy individuals” are those who have achieved a normative process of individuation and as such, remain individually bounded and fundamentally impermeable to others. This “molecular” model of an interior, individual personhood, Obadia shows, is a precondition for acceptable “healthy” intimacy in late liberal North America (2020: 1397). Neuroreductive conceptualizations of subjective and embodied experience (including distress) in terms of brain-based sensory difference overlap with this bounded, impermeable model of the self. As Francisco Vidal and Fernando Ortega have argued in their book, *Being Brains: The Making of the Cerebral Self* (2017), contemporary cerebral subjecthood is haunted by a possessive individualism (the self as one’s private property) long extant in liberal Western thought. Moreover, such subjecthood locates, or seals, human capacities and identity in the brain, thus filtering out external, relational dynamics, social events and “the messy ‘phenomenological, embodied, and affective dimensions of human experience’” (Vidal and Ortega quoted in Lunbeck, 2018). In such an ideological context, our readings of embodied experience can minimize relational experiences and capacities, and individualized, medicalized readings of difference that link neurodivergent children’s behavior to individualized interior conditions encapsulated in the brain (e.g., “sensory issues”) easily make sense. At the same time, “personal space,” which children like Sam often trespass comes to be both emphasized and policed. Indeed, in our first interview, V described Sam’s difference around personal boundaries as her “...biggest fear, I don’t want him to impinge on people.”

In Obadia’s case, it is the intimate proximity of others that threatens subjects’ sense of inviolability. In V’s narrations, it is haptic, visual, and spoken strategies of care; family life in a small apartment; labors of attunement oriented toward better care; and media that have led to her questioning of a self-contained, individualized, neurosensory reading of her son’s experience. These practices and experiences have thus enhanced her recognition of the extent of Sam’s capacity to be affected by (or permeable to) others in social life, or by environments or physical sensations. As I have argued, her reworked understanding constitutes an ethical gift of self-transformation, but I suggest that it may also hold broader political, and perhaps practical, value. For one, it reveals contemporary, popular discourses of sensory difference to be an inadequate and stereotyped account of children’s anxious, embodied suffering—something those providing supportive care to neurodivergent children, particularly those with “co-morbid” mental health challenges and diagnoses might productively entertain. Further, her reappraisal rejects a tendency of neurodiversity discourse to present neurodivergent people’s brains homogeneously as the same kind of “different” (cf. Silverman, 2018 comment on Ortega and Vidal (2017)).

Second, V’s emergent perception demonstrates how experimental practices of asymmetrical disability care can, in visceral ways, work against a late liberal North American cultural tendency to fetishize individual boundedness and interiority, and reveal instead our permeability to others. In the context of middle-class parental care for neurodivergent children in the United States, such a challenge to individual boundedness and interiority may take root as caretakers develop, as V did, attuned sensitivity to the cared-for others’ embodied experience. Further, it may gain salience as caretakers and cared-for ‘others share experiences across bodies, and incorporate into care particular attentiveness to how children are affected by their ties to others, social life and environments, and bodily sensations. It is important, in recounting a one-sided narrative of caretaking from the perspective of the caretaker, to note the great limitations of privileging the caretaker’s perspective, and the always partial and uneven nature of intersubjective encounters. Nonetheless, V’s narration of care reveals an ethics of care in-the-making forged through relationality across bodies and within a social world. As such, it exposes deep linkages between “care at its most intimate and broader socio-political concerns” (Buch, 2015: 283).

## Conclusion

Parents’ caretaking narratives focused on the care of neurodivergent children offer an opportunity to learn about the relation between care practices and the caretaker’s shifting ethical perspectives concerning the embodied experience of the child, and how to provide better care. In particular, parents’ labors of attunement, painstaking observation of children, and attempts to offer effective care, particularly in moments of a child’s embodied distress, reveal care to be an experimental, morally uncertain, and perceptively dynamic process. This analysis has attended to enactments of care at the individual scale via one mother’s narration of her lived caretaking past and present. In doing so, it has highlighted how forms of care and caretaking experience can engender for the caretaker a sense of intersubjective understanding that, in turn, shapes affective orientation toward the cared-for child and the caretaker’s practices and beliefs about care. Intimate moments of shared embodied experience and intercorporeal connection, and consistently attuned observation of a cared-for child at different times and within different environments are central to this process. So are caretakers’ engagements with relevant cultural resources. Additionally, this account has illuminated a transformation in the caretaker’s understanding of the child’s embodied experience of distress and excitement; the caretaker stopped attributing such states to “sensory issues,” and came to appreciate them in terms of “anxiety,” understood to be socially, emotionally, and physically constituted in relation to others and environments as well as bodily feelings. This perceptual shift constitutes a practice-engendered ethical gift of self-transformation. The gift of this new perspective enables care more specifically tailored to the embodied experience of a neurodivergent child. At the same time, it resists doxic and generalized understandings of neuroatypical experience of anxiety as solely an expression of interior, individualized neurosensory difference. As such, it counters a reading of embodied experience and difference that circulates in North American care for neurodivergent childhood, and articulates with late liberal notions of impermeable, bounded individual personhood.

### *V and Sam are Pseudonyms

This research did not receive any specific grant from funding agencies in the public, commercial, or not-for-profit sectors. Sabbatical funding from my institution supported data collection in an earlier phase of the project.

## Data Availability

This research has received IRB approval via the Montclair State University IRB Review Board. The paper draws on video interviews and other interview transcripts with research subjects whose confidentiality is protected. As such, the ethnographic data provided and analyzed in the paper is not publicly available.
